# In Situ Metabolomics Expands the Spectrum of Renal Tumours Positive on ^99m^Tc-sestamibi Single Photon Emission Computed Tomography/Computed Tomography Examination

**DOI:** 10.1016/j.euros.2020.11.001

**Published:** 2020-11-27

**Authors:** Thomas Papathomas, Antonios Tzortzakakis, Na Sun, Franziska Erlmeier, Annette Feuchtinger, Kiril Trpkov, Alina Bazarova, Alexandros Arvanitis, Wanzhong Wang, Bela Bozoky, Georgia Kokaraki, Rimma Axelsson, Axel Walch

**Affiliations:** a Institute of Metabolism and Systems Research, University of Birmingham, Birmingham, UK; b Gloucestershire Cellular Pathology Laboratory, Cheltenham General Hospital, Gloucestershire Hospitals NHS Foundation Trust, Cheltenham, UK; c Division of Radiology, Department for Clinical Science, Intervention and Technology (CLINTEC), Karolinska Institutet, Stockholm, Sweden; d Medical Radiation Physics and Nuclear Medicine, Section for Nuclear Medicine, Karolinska University Hospital, Huddinge, Stockholm, Sweden; e Research Unit Analytical Pathology, German Research Center for Environmental Health, Helmholtz Zentrum München, Munich, Germany; f Institute of Pathology, University Hospital Erlangen, Friedrich-Alexander-University Erlangen-Nürnberg, Erlangen, Germany; g Institute of Pathology, Technical University Munich, Munich, Germany; h Department of Pathology and Laboratory Medicine, Alberta Precision Labs, Cumming School of Medicine, University of Calgary, Calgary, Canada; i Institute for Biological Physics, University of Cologne, Cologne, Germany; j Department of Clinical Pathology and Cytology, Karolinska University Hospital, Huddinge, Stockholm, Sweden; k Department of Oncology-Pathology, Karolinska University Hospital, Stockholm, Sweden

**Keywords:** 99mTc-sestamibi SPECT/CT, Renal tumour/in situ metabolomics

## Abstract

**Background:**

Definite noninvasive characterisation of renal tumours positive on ^99m^Tc-sestamibi single photon emission computed tomography/computed tomography (SPECT/CT) examination including renal oncocytomas (ROs), hybrid oncocytic chromophobe tumours (HOCTs), and chromophobe renal cell carcinoma (chRCC) is currently not feasible.

**Objective:**

To investigate whether combined ^99m^Tc-sestamibi SPECT/CT and in situ metabolomic profiling can accurately characterise renal tumours exhibiting ^99m^Tc-sestamibi uptake.

**Design, setting, and participants:**

A tissue microarray analysis of 33 tumour samples from 28 patients was used to investigate whether their in situ metabolomic status correlates with their features on ^99m^Tc-sestamibi SPECT/CT examination. In order to validate emerging data, an independent cohort comprising 117 tumours was subjected to matrix-assisted laser desorption/ionisation mass spectrometry imaging (MALDI MSI).

**Outcome measurements and statistical analysis:**

MALDI MSI data analysis and image generation were facilitated by FlexImaging v. 4.2, while k-means analysis by SCiLS Lab software followed by R-package CARRoT analysis was used for assessing the highest predictive power in the differential of RO versus chRCC. Heatmap-based clustering, sparse partial least-squares discriminant analysis, and volcano plots were created with MetaboAnalyst 3.0.

**Results and limitations:**

We identified a discriminatory metabolomic signature for ^99m^Tc-sestamibi SPECT/CT–positive Birt-Hogg-Dubè–associated HOCTs versus other renal oncocytic tumours. Metabolomic differences were also evident between ^99m^Tc-sestamibi–positive and ^99m^Tc-sestamibi–negative chRCCs, prompting additional expert review; two of three ^99m^Tc-sestamibi–positive chRCCs were reclassified as low-grade oncocytic tumours (LOTs). Differences were identified between distal-derived tumours from those of proximal tubule origin, including differences between ROs and chRCCs.

**Conclusions:**

The current study expands the spectrum of ^99m^Tc-sestamibi SPECT/CT–positive renal tumours, encompassing ROs, HOCTs, LOTs, and chRCCs, and supports the feasibility of in situ metabolomic profiling in the diagnostics and classification of renal tumours.

**Patient summary:**

For preoperative evaluation of solid renal tumours, ^99m^Tc-sestamibi single photon emission computed tomography/computed tomography (SPECT/CT) is a novel examination method. To increase diagnostic accuracy, we propose that ^99m^Tc-sestamibi–positive renal tumours should be biopsied and followed by a combined histometabolomic analysis.

## Take Home Message

Our study provides novel molecular insights into renal neoplasia, and supports the feasibility of integrated in situ metabolomic profiling for the diagnostics and classification of renal tumours. The results of this study suggest that renal tumours positive on ^99m^Tc-sestamibi single photon emission computed tomography/computed tomography should be biopsied and analysed in an integrated fashion to inform clinical management.

## 1 Introduction

Recent advances in genomics and molecular genetics have provided novel insights into renal tumorigenesis refining the molecular classification of renal cancer [1]. In addition to providing a better understanding of the molecular landscape of major renal cell carcinoma (RCC) subtypes, a pan-genomic study from The Cancer Genome Atlas Research Network has led to a more accurate definition of their biological behaviour [2]. A subset of metabolically divergent RCCs was identified, displaying a distinct metabolic expression associated with extremely poor survival [2]. This has confirmed the recently documented correlation of metabolic expression subtypes and patient survival across various and diverse malignancies [3].

Imaging with ^99m^Tc-sestamibi single photon emission computed tomography/computed tomography (SPECT/CT) was recently introduced for preoperative RCC diagnosis by dividing solid renal tumours into positive or negative ones on the basis of tracer uptake [4]. As a mitochondrial agent, sestamibi uptake correlates with different mitochondrial content and variable multidrug resistance pump expression that renal tumours exhibit [5]. The ^99m^Tc-sestamibi–positive tumours exhibiting increased ^99m^Tc-sestamibi uptake are more likely to be benign or of low malignant potential, whereas the ^99m^Tc-Sestamibi–negative counterparts appear to have malignant characteristics. The latter group could be considered for surgery, while the former could potentially be managed conservatively by active surveillance utilising long follow-up with or without renal biopsy [6].

Technological advances of molecular imaging and pathology are expected to reshape modern medicine. For example, a diagnostic differentiation of renal oncocytomas, a benign tumour, from malignant renal tumours appears promising on imaging grounds. Nevertheless, false-positive and false-negative results on ^99m^Tc-sestamibi SPECT/CT confound certain aspects of its clinical utility. Herein, we investigate the in situ metabolomic status of a ^99m^Tc-sestamibi SPECT/CT–examined cohort of renal tumours and propose an integrated approach that combines molecular imaging and in situ metabolomic profiling to better characterise renal neoplasia and potentially improve patient management.

## 2 Patients and methods

### 2.1 Case selection

Forty-two out of the 50 patients who were included in the current study participated previously in an institutional review board–approved prospective study to investigate imaging characteristics of solid renal tumours (T1; ≤7 cm) using ^99m^Tc-sestamibi SPECT/CT [6]. The results of the investigation were reported in two consecutive studies [7], [8]. A complimentary approval by the Regional Ethical Review Board (2018/1626) and the Stockholm Biobank (Bbk 2082) along with a newly requested informed consent form was obtained. This study was approved by the Regional Ethical Review Board and the local Radiation Safety Committee (reference number 2015/923-31/4). Consent was acquired from all patients who participated in our study.

Haematoxylin and eosin (HE) slides as well as immunoslides from biopsy and resection specimens were retrieved from the archives of Karolinska University Hospital in Huddinge and assessed anonymously. Slides were independently reviewed by two consultant histopathologists (T.P. and W.W.) in a blinded manner in order to reach a consensus on the final diagnosis. This diagnosis was utilised as the gold standard with which all hybrid molecular imaging and in situ metabolomic data were compared.

Eleven patients were excluded from the final analysis owing to a lack of or a very limited amount of tumour bioptic tissue within the FFPE blocks, given extensive immunohistochemical work-up on diagnostic grounds and/or lack of informed consent. The initial evaluated cohort included 45 tumours from 39 patients, encompassing nine renal oncocytomas (ROs), five hybrid oncocytic chromophobe tumours (HOCTs), eight chromophobe RCCs (chRCCs), eight clear cell RCCs (ccRCCs), nine papillary RCCs (pRCCs), two clear cell pRCCs, and one case each of papillary adenoma, lymphoma, angiomyolipoma, and metanephric adenoma.

### 2.2 TMA construction and MALDI MSI analysis

To investigate the in situ metabolomic status, tumour samples from biopsy and resection specimens were arranged in a tissue microarray analysis (TMA) format using a semiautomated tissue arrayer MiniCore. For each tumoural case, representative areas were selected and marked on an HE-stained slide. Accordingly, three cores (for resections) and/or one core (for biopsies) with a diameter of 1 mm were extracted from the “donor” block and brought into the “recipient” paraffin block. To validate emerging data, we used part of a previously published cohort [9], comprising 117 tumours: 59 ROs and 58 chRCCs. Sections of 4 μm were subsequently cut from TMA blocks.

Matrix-assisted laser desorption/ionisation (MALDI) mass spectrometry imaging (MSI) analysis was performed at the Research Unit Analytical Pathology (Helmholtz Zentrum München), as described previously by Ly et al [10]. MALDI time of flight (TOF) MSI measurements were carried out with 60 μm lateral resolution over the analysed mass range of *m*/*z* 100–1000 in the negative reflector ion mode. A Smartbeam-II Nd:YAG laser was equipped with a frequency of 100 Hz. The sampling rate of 2.0 GS/s and a total of 200 laser shots were used for each measurement position. MALDI Fourier transform ion cyclotron resonance (FT-ICR) MSI measurements were performed over the mass range of *m*/*z* 50–1000 in the negative ion mode. For each measurement position, 100 laser shots were accumulated using a Smartbeam-II Nd:YAG (355 nm) laser operating at a frequency of 500 Hz.

Following MALDI MSI analysis, the matrix was removed with 70% ethanol and stained with HE using a fully automated tissue stainer (Tissue Stainer TST 44C; MEDITE, Leica, Nussloch, Germany). Slides were subsequently scanned utilising a MIRAX DESK digital slide-scanning system (Carl Zeiss MicroImaging, Gottingen, Germany). To spatially relate the signal intensities to histopathological features in individual tissue spots, digital images were coregistered to respective MSI data using FlexImaging v. 4.2 and SCiLS Lab version 2017 (Bruker Daltonic, Bremen, Germany). Only signals that are typically colocalised with neoplastic cells were classified.

Twelve cases were further excluded as either sufficient tissue was not present in the TMA sections due to technical issues given the limited bioptic material (six biopsy cases: two ccRCCs, two HOCTs, one chRCC, and one pRCC) or the cases were under-represented in numbers (ie, three samples required per tumour category for statistical analysis; six cases: two clear cell pRCCs, one papillary adenoma, one lymphoma, one angiomyolipoma, and one metanephric adenoma). Hence, the first set was reduced to 33 tumours: nine ROs, eight pRCCs type 1, seven chRCCs, six ccRCCs, and three HOCTs.

### 2.3 Data processing

Data analysis and image generation were facilitated by FlexImaging v. 4.2. The total ion current of each spectrum was used for signal intensity normalisation. Histology-guided regions of interests (ROIs) were annotated to generate average mean spectra. Global spectra of TOF and FT-ICR were exported from FlexImaging and SCiLS Lab. Peak picking on the average mean spectra of the defined ROIs was conducted in mMass Version 5.5.0 utilising the Savitzky-Golay algorithm and an *s*/*n* ratio of 2. Peaks of TOF and FT-ICR were matched with a window of 0.2 Dalton.

Metabolites were annotated by matching accurate mass with databases (ion mode: negative; adduct type: [M-H], [M-H-H2O], [M + Na-2 H], [M + Cl], and [M + K-2 H]; mass accuracy ≤4 ppm; Human Metabolome Database [HMDB]) [11]. Heatmap-based clustering analysis, component analysis, volcano plots, and pathway analysis were performed with MetaboAnalyst 3.0 [12] and KEGG database [13].

## 3 Results

### 3.1 Clinicopathological characteristics of ^99m^Tc-sestamibi SPECT/CT–analysed renal tumours and initial metabolomic data acquisition

The examined cohort comprised a total of 33 renal tumours from 28 patients (Table 1). Seven of nine ROs were positive, while two of nine were negative on the ^99m^Tc-sestamibi SPECT/CT examination. All three HOCTs identified in a female patient with verified Birt-Hogg-Dubè (BHD) syndrome were also positive. The BHD patient had a germline *FLCN* heterozygous mutation (c.779 + 1G > T), repeated episodes of pneumothorax, and cutaneous basal cell carcinomas. Three of seven chRCCs exhibited a positive ^99m^Tc-sestamibi uptake, whereas the remaining four chRCCs were negative (Table 2). All evaluated ccRCCs (6/6) and pRCCs (8/8) were negative on the ^99m^Tc-sestamibi SPECT/CT examination.

**Table 1:** Clinicopathological characteristics of patients with renal tumours assessed by molecular imaging and metabolomics

Tumour type	No. of patients	No. of tumours	Female/male ratio	Age (yr, mean)	Tumour size (mm, mean)
RO	6	9	0/6	68.8	41.0
HOCT	1	3	1/0	60.0	13.0
chRCC	7	7	3/4	64.6	31.9
ccRCC	6	6	1/5	69.3	33.5
pRCC (type1)	8	8	1/7	59.9	21.3

ccRCC: = clear cell renal cell carcinoma; chRCC = chromophobe renal cell carcinoma; HOCT = hybrid oncocytic chromophobe tumour; pRCC = papillary renal cell carcinoma; RO = renal oncocytoma.

**Table 2:** Visual evaluation of ^99m^Tc-sestamibi uptake on 33 solid renal tumours from 28 patients

Tumour type	No. of renal tumours	^99m^Tc-sestamibi positive, *n* (%)	^99m^Tc-sestamibi negative, *n* (%)
RO	9	7 (78)	2 (22)
HOCT	3	3 (100)	0
chRCC	7	3 (43)	4 (57)
ccRCC	6	0	6 (100)
pRCC (type 1)	8	0	8 (100)

ccRCC: = clear cell renal cell carcinoma; chRCC = chromophobe renal cell carcinoma; HOCT = hybrid oncocytic chromophobe tumour; pRCC = papillary renal cell carcinoma; RO = renal oncocytoma.

Overall, approximately 770 individuals’ MS peaks per pixel within the mass range of *m*/*z* 100–1000 could be resolved within the tissue examined, while 319 metabolites were annotated through the HMDB.

### 3.2 Hierarchical clustering analysis of metabolomic data segregates positive BHD-associated HOCTs and distinguishes LOTs from classic chRCCs

**Fig. 1:**
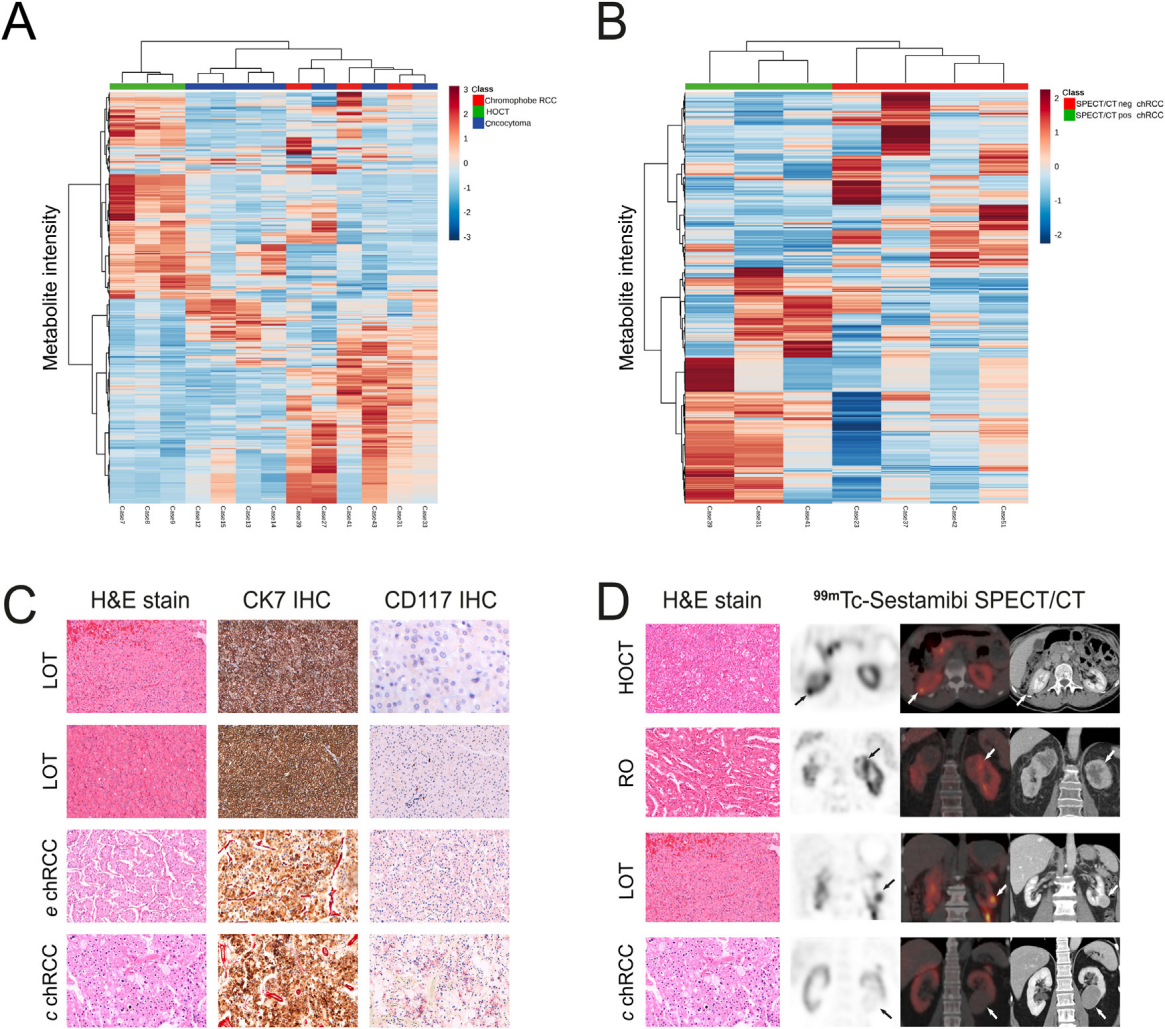
Metabolomic data analysis segregates ^99m^Tc-sestamibi SPECT/CT–positive BHD-associated HOCTs and distinguishes SPECT/CT–positive LOTs from classic chRCCs. (A) Unsupervised clustering analysis based on discriminative metabolites (*n* = 460) depicting a clear separation between ^99m^Tc-sestamibi SPECT/CT–positive BHD-associated HOCTs and other ^99m^Tc-sestamibi SPECT/CT–negative renal oncocytic neoplasms. (B) Heatmap of top 420 m/z values highlights different m/z expression patterns in SPECT/CT photophilic chRCCs versus photopenic counterparts. (C) These in situ metabolomic differences prompted a pathological evaluation of all chRCCs: histopathological features of three ^99m^Tc-sestamibi SPECT/CT–positive chRCCs (case numbers 41, 31, and 39; top to bottom), which were amended to LOTs (case numbers 41 and 31) and eosinophilic chRCC (case number 39) upon expert review. A ^99m^Tc-sestamibi SPECT/CT–negative classic chRCC (case number 51) is also included in the panel (bottom). (D) Histological features and hybrid molecular imaging (scintigraphic, SPECT/CT, and CT study) of three ^99m^Tc-sestamibi SPECT/CT–positive cases: HOCT (case number 8: axial view of a 13-mm tumour on the dorsal aspect of the right kidney exhibiting ^99m^Tc-sestamibi uptake), RO (case number 14: coronal view of a 60-mm tumour with a necrotic component on the upper pole of the left kidney exhibiting ^99m^Tc-sestamibi uptake), and LOT (case number 41: coronal view of a 28-mm tumour on the lower pole of the left kidney exhibiting ^99m^Tc-sestamibi uptake), as well as a ^99m^Tc-sestamibi SPECT/CT–negative classic chRCC (case number 51: coronal view of a 64-mm tumour on the medial aspect of the lower pole of the left kidney without ^99m^Tc-sestamibi uptake; top to bottom). BHD = Birt-Hogg-Dubè; chRCC = chromophobe renal cell carcinoma; *c* chRCC = classic-type chRCC; CT = computed tomography; *e* chRCC = eosinophilic variant of chRCC; H&E = haematoxylin and eosin; HOCT = hybrid oncocytic chromophobe tumour; IHC = immunohistochemistry; LOT = low-grade oncocytic tumour; RCC = renal cell carcinoma; RO = renal oncocytoma; SPECT = single photon emission computed tomography.

Unsupervised hierarchical clustering analysis identified a discriminatory metabolomic signature for positive BHD-associated HOCTs. This is in accordance with the recent molecular evidence suggesting that an HOCT represents an entity with genomic features intermediate between an RO and a chRCC [14]. All HOCTs as well as the three ROs arising in one patient were subclustered together (Fig. 1A). Metabolomic differences were also found between positive and negative chRCCs (Fig. 1B), prompting an expert review of the morphological and immunohistochemical features of the set of cases initially considered “chRCCs”. Two of three positive chRCCs were reclassified upon review as “low-grade oncocytic tumours” (LOTs). A LOT is an emerging renal entity with morphological features overlapping those of an RO and a chRCC that demonstrates a CK7 pos./CKIT neg. immunoprofile, and lacks the multiple chromosomal losses typically seen in chRCCs [15]. The third positive chRCC was classified as an eosinophilic chRCC upon review (Fig. 1C). This approach reduced the total number of positive chRCCs to one, and hence further comparison of positive ROs versus chRCCs was precluded. However, the tumour spectrum of renal neoplasms exhibiting uptake on ^99m^Tc-sestamibi SPECT/CT examination was expanded (Fig. 1D), with potential implications for clinical management.

### 3.3 Metabolic alterations in RCC subtypes with regard to the presumed origin

**Fig. 2:**
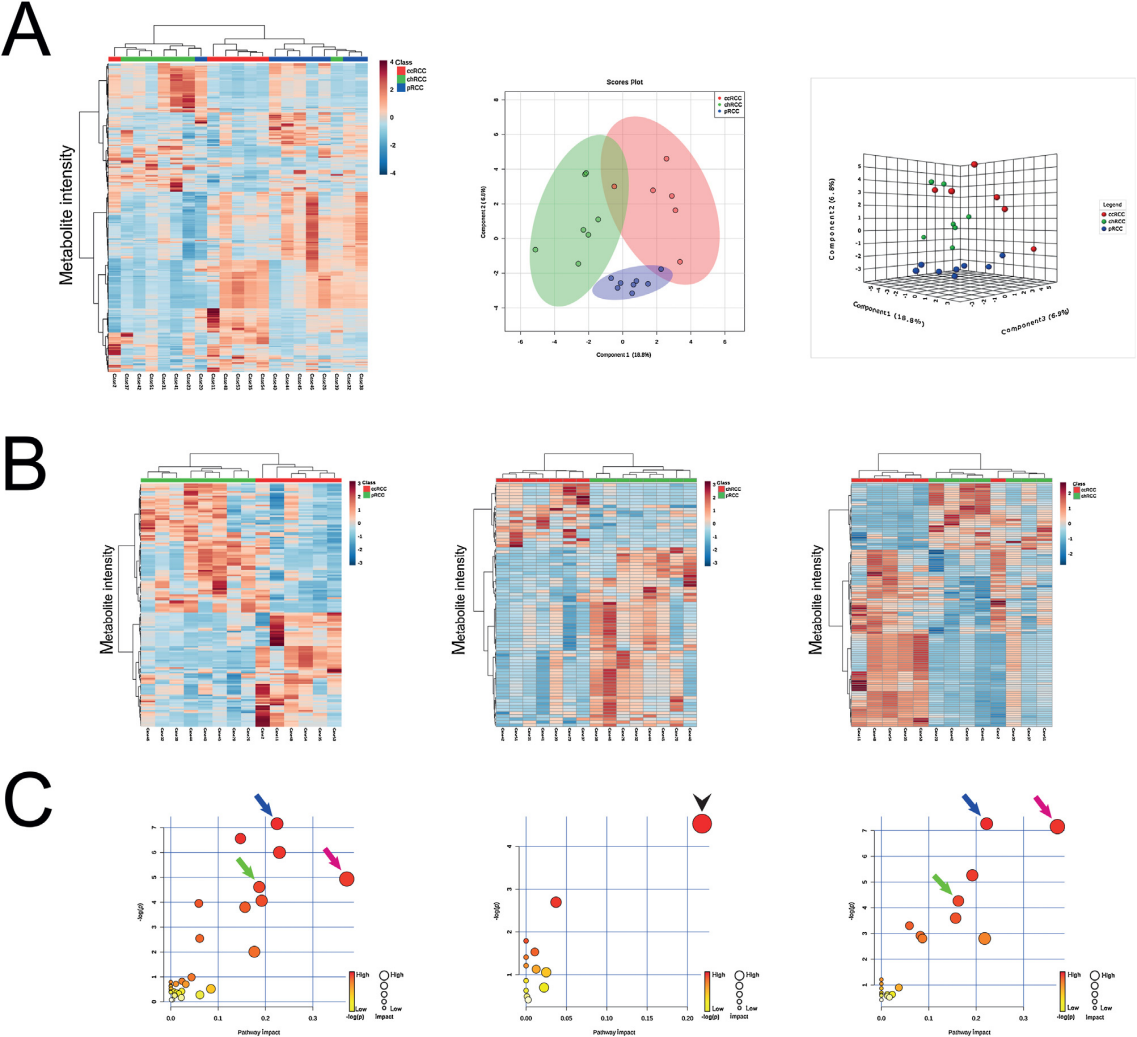
In situ metabolomic alterations in ^99m^Tc-sestamibi SPECT/CT-examined RCC subtypes. (A) Heatmap of top 370 differentially intense m/z values highlights different m/z expression patterns in ccRCCs, pRCCs and chRCCs (left), while sPLSDA plots (middle and left) distinguish between these RCC subtypes. (B) Unsupervised clustering analysis illustrating a clear separation between pRCCs and ccRCCs (*n* = 160; left) and pRCCs and chRCCs (*n* = 80; middle), with only one misclassified case between ccRCCs and chRCCs (*n* = 110; right). (C) Common modulated pathways in the distinction of ccRCCs versus pRCCs (left) and ccRCCs versus chRCCs (right) impacting fructose and mannose metabolism (blue arrow), galactose metabolism (green arrow), as well as aminosugar and nucleotide sugar metabolism (pink arrow), whereas glycerophospholipid metabolism (black arrowhead) is highlighted in the distinction of chRCCs versus pRCCs (middle). Metabolic pathways are represented as circles according to their scores from enrichment (*y* axis) and topology analyses (*x* axis). The colour of circles indicates the statistical significance of the overall metabolic changes within the pathway, and circle diameter represents the relative impact of differential metabolites within the pathway, as indicated. ccRCC = clear cell renal cell carcinoma; chRCC = chromophobe renal cell carcinoma; CT = computed tomography; pRCC = papillary renal cell carcinoma; RCC = renal cell carcinoma; SPECT = single photon emission computed tomography; sPLSDA = sparse partial least-squares discriminant analysis.

Similar to other studies on metabolomic and gene expression profiling [16], we confirmed the differences between the distal nephron-derived tumours (ie, chRCCs) from those originating from the proximal tubules (ie, ccRCCs and pRCCs), and highlighted others within the same subgroups. Annotated metabolites emerging from heatmaps and volcano plots responsible for the metabolic differentiation were further investigated using a pathway enrichment analysis. Modulated biochemical pathways in the distinction of ccRCCs versus pRCCs, ccRCCs versus chRCCs, and pRCCs versus chRCCs are depicted in Fig. 2.

### 3.4 Metabolomic differences between ROs and chRCCs utilising hierarchical clustering and k-means analysis

**Fig. 3:**
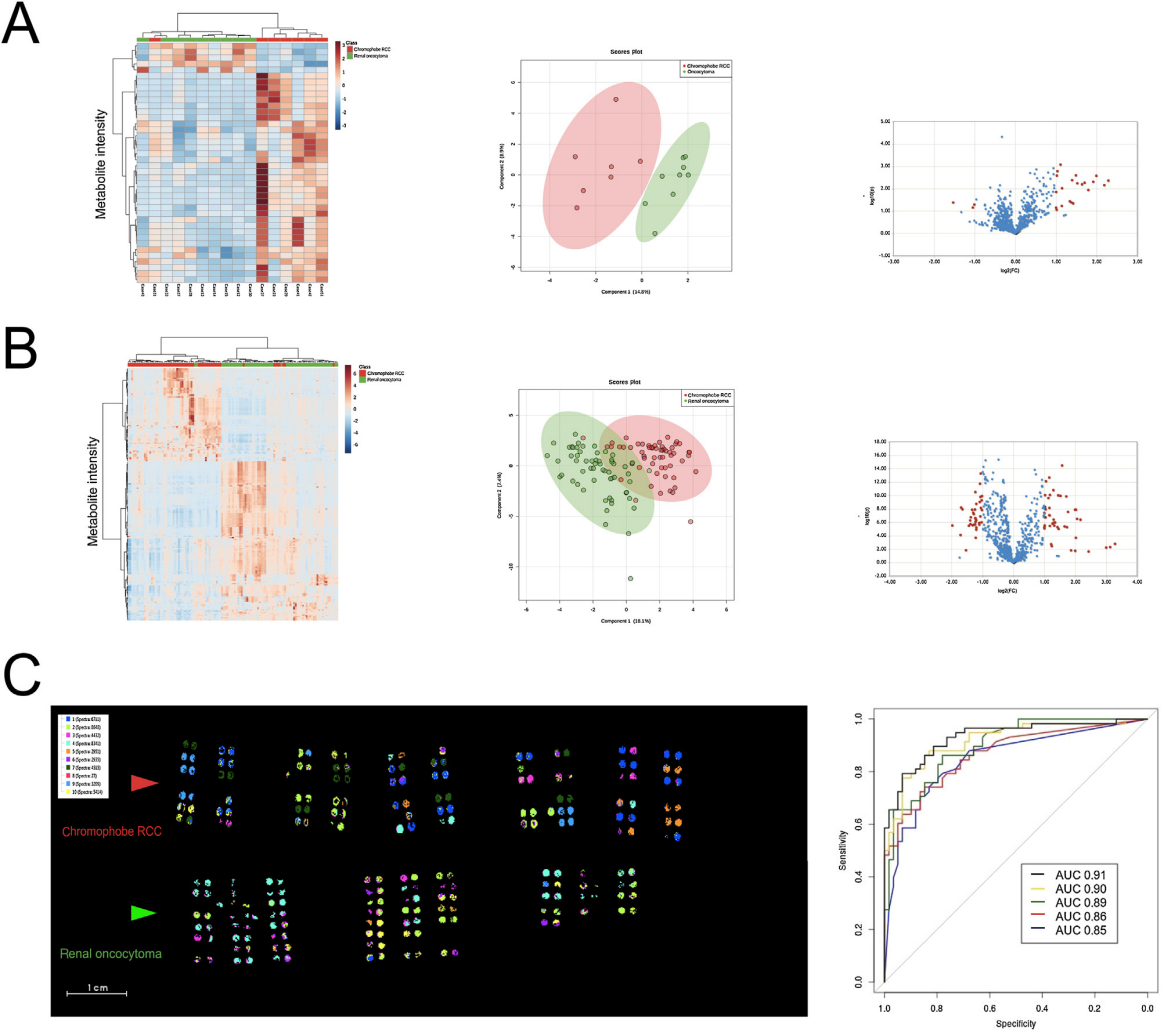
In situ metabolomic differences between renal oncocytomas and chromophobe RCCs utilising hierarchical clustering and k-means analysis. Heatmaps depicting unsupervised clustering based on discriminative metabolites (Fig. 3A: *n* = 40; Fig. 3B: *n* = 230) and sPLSDA plots (left and middle) revealing separation of RO and chRCC cases as well as volcano plots (right) exhibiting differential m/z values in RO versus chRCC cases in the (A) ^99m^Tc-sestamibi SPECT/CT–examined set and (B) validation set. With regard to volcano plots, both fold changes (FCs, *x* axis; threshold 2) and *p* values (*y* axis; threshold 0.01) are log-transformed. Red dots represent significant metabolites. The further the red dot from the (0.0), the more significant the feature in the distinction of RO versus chRCC. (C) Mass spectra and ion distribution maps based on ten clusters of metabolites as generated by k-means analysis utilising SCiLS Lab software; red and green arrows indicate chRCCs and ROs of the validation cohort, respectively (left). ROC curves corresponding to the best predictive model based on separating the metabolites into two (blue), four (red), six (green), eight (yellow), and ten (black) clusters. Note a general trend of increasing predictive power with the number of clusters, and hence the best predictive power is exhibited by the model of ten clusters (right). AUC = area under the curve; chRCC = chromophobe renal cell carcinoma; CT = computed tomography; RCC = renal cell carcinoma; RO = renal oncocytoma; SPECT = single photon emission computed tomography; sPLSDA = sparse partial least-squares discriminant analysis.

Using high-resolution MALDI-FT-ICR MSI, we previously identified metabolomic signatures, based on the top 50 differentially intense m/z values, which accurately distinguished ROs from chRCCs [17]. Herein, a MALDI-TOF MSI analysis of the first set yielded similar results with one chRCC misclassified (one out of 16; 6.25%; Fig. 3A), while two ROs and eight chRCCs were misclassified upon validation (ten out of 117; 8.54%; Fig. 3B).

Following an alternative approach to investigate the predictability of ROs versus chRCCs, we performed k-means analysis on the validation set utilising SCiLS Lab software. Metabolites were separated into two and up to ten clusters in an effort to identify the combination of clusters yielding the highest predictive power using R-package CARRoT [18]; this is a predictive software tool that performs model selection as per the best subset regression by using the “one in ten” rule.

The cluster signal and its percentage values were used as predictive variables with CARRoT run on each of the splits into a separate cluster. In each of the nine scenarios (ie, two to ten clusters), we set the number of cross-validations to 1000, by dividing the dataset into training (90% of the data) and testing (10% of the data) sets. For each cluster separation, we utilised the same 1000 partitions in order to facilitate the comparison of the predictive power. The latter approach was quantified by the area under the receiver operator curve (AUROC) with the corresponding average AUROC values based on 1000 cross-validations, as follows: 0.85 (two clusters), 0.88 (three clusters), 0.86 (four clusters), 0.89 (five clusters), 0.89 (six clusters), 0.90 (seven clusters), 0.90 (eight clusters), 0.91 (nine clusters), and 0.91 (ten clusters; Fig. 3C)

Clusters with the highest predictive power contained several m/z values of a very high level of intensity (ie, several outliers). In an effort to identify those values for each cluster, k-means analysis was performed by splitting each cluster into two parts. The resultant silhouette score was no less than 0.96, indicating a very good separation. The m/z values 92.898, 94.895, 96.924, 193.078, 194.067, and 229.076 exhibited high intensity simultaneously in all the aforementioned clusters. Additional research is warranted to further validate the identity of these m/z values.

## 4 Discussion

In this work, we have provided an in situ metabolomic analysis of renal tumours previously analysed by molecular imaging. This approach expands the spectrum of ^99m^Tc-sestamibi SPECT/CT–positive renal tumours encompassing ROs, HOCTs, LOTs, and chRCCs, and supports combined diagnostics utilising molecular imaging and histometabolomic profiling. The current study further substantiates the value of metabolomics with regard to the molecular profiling of renal neoplasms [19].

Our findings also support the feasibility of a metabolomic profiling in the subclassification of renal tumours that is currently based on a morphology-based diagnostics. In situ metabolomics provides a promising tool particularly in assessing renal oncocytic neoplasms (eg, RO vs chRCC differential) and when potentially assessing limited core biopsy specimens. According to a systematic review and meta-analysis [20], core biopsy may be unreliable in establishing a definitive diagnosis of oncocytoma. Challenging cases within the oncocytic spectrum of renal neoplasia are in fact encountered frequently, including an intermediate diagnostic category [21], cases exhibiting low-/high-grade oncocytic morphology [15], [22], and other less common eosinophilic tumours, including an epithelioid angiomyolipoma, SDH-deficient RCC [23], and FH-deficient RCC [24].

Overlapping and/or misclassified cases in 2-D score plot(s) and heatmap(s) also highlight the need to integrate this metabolomic data into a proper pathological context. This is consistent with previous studies demonstrating either misclassified ccRCCs as chRCCs [16] or overlapping chRCCs and oncocytomas [25]. Although the former was suggested as a potential erroneous pathological interpretation [16], this was not the case in our series, possibly reflecting metabolomic similarities. With regard to the latter, we observed one positive chRCC within the RO subgroup (Fig. 3A) and two ROs within the chRCC subgroup (Fig. 3B). This likely reflects the limitations of the current classification and the expanding spectrum of renal oncocytic neoplasia [21], [22].

As a matter of fact, two positive cases initially considered chRCCs (eosinophilic type) clustered together and separately from the negative classic chRCCs; both were reclassified as LOTs upon expert review (Fig. 1B and 1C). This newly proposed emerging renal entity is characterised by consistent morphological traits that overlap ROs and chRCCs, CK7 pos./c-Kit neg. immunoprofile, absence of multiple chromosomal losses and gains, and indolent clinical behaviour [15]. Three BHD-associated HOCT cases also exhibited a distinct metabolomic profile (Fig. 1A), further reinforcing the concept that HOCTs may represent a unique renal entity [14] and not a chRCC subtype/variant, according to the current WHO classification [26]. Whether this discriminatory metabolomic signature could directly be attributed to a specific genetic make-up associated with germline *FLCN* mutations remains to be investigated further [27].

The current study expands the ontogeny considerations based on the evidence emerging from molecular genetic studies. We confirmed the recent findings supporting distinctive metabolomic signatures in histogenetically related oncocytic tumours and RCC subtypes [28]. Priolo et al [25] utilised MS-based metabolomics, and demonstrated both similarities and differences between chRCCs and ROs with a clear separation in a principal component analysis scatterplot of the log_2_ ratio of metabolite levels in tumours to matched normal samples. This has also been corroborated by an untargeted in situ metabolomic approach based on MALDI-FT-ICR MSI, which clearly displayed a metabolomic distinction between ROs and chRCCs [28]. Schaeffeler et al [16] provided evidence that tumours originating from proximal nephron could be differentiated from distal nephron-derived tumours utilising targeted metabolomic/lipidomic analyses, while Steurer et al [29] identified subtype-specific metabolomic differences in proximal-derived tumours on MALDI-TOF MSI.

Our study has several limitations as the number of cases investigated using both ^99m^Tc-sestamibi SPECT/CT and MALDI-MSI was rather small with variable representation of tumour entities. To exemplify, neither a pRCC type II nor an HOCT was included outside of the BHD context (ie, sporadic or associated with renal oncocytosis).

Another consideration refers to the preselected spectrum of MS peaks and annotation of *m*/*z* species, that is, the range of metabolites, captured by MALDI-TOF MSI analysis. In fact, only a small number of biomarkers are actually shared between different analytical platforms and biological specimens [30]. Towards metabolic differentiation between chRCCs and ROs, urinary metabolomics highlighted citrate, carnitine, transaconitate, succinate, and π-methylhistidine [31], while tissue metabolomics revealed glycolysis and pentose phosphate pathway intermediates along with certain gamma-glutamyl amino acids as differential metabolites [25].

Hence, a prospective study is warranted at a large scale encompassing sporadic and hereditary forms of renal neoplasia, with a distribution reflective of their prevalence in clinical practice. That being said, specific in situ metabolomic signatures per tumour type shall be validated subsequently in an independent set and compared with current histopathological assessment of renal core biopsies. If not superior, complementary aspects of in situ metabolomics to current practice with regard to renal oncocytic tumours positive on ^99m^Tc-sestamibi SPECT/CT examination should be explored and further specified. As intratumour genetic and metabolic heterogeneity has been demonstrated in renal cancer [32], in keeping with the phenotypic intratumour heterogeneity utilising MSI [33], further studies are additionally required to investigate whether heterogeneity might confound this combined method.

## 5 Conclusions

The present study provides novel molecular insights into renal neoplasia and supports the feasibility of an integrated in situ metabolomic profiling for the diagnostics and classification of renal tumours. The results of this study suggest that renal tumours positive on ^99m^Tc-estamibi SPECT/CT should be biopsied and analysed in an integrated fashion to inform clinical management. This approach establishes a foundation for future studies to define more accurately in situ metabolomic signatures of various renal tumour histologies and genotypes.

  ***Author contributions*:** Antonios Tzortzakakis had full access to all the data in the study and takes responsibility for the integrity of the data and the accuracy of the data analysis.

  *Study concept and design*: Tzortzakakis, Papathomas, Axelsson, Walch.

*Acquisition of data*: Tzortzakakis, Papathomas, Sun, Feuchtinger, Erlmeier, Trpkov.

*Analysis and interpretation of data*: Tzortzakakis, Papathomas, Sun, Bazarova, Trpkov, Bozoky, Wang, Arvanitis, Axelsson, Walch.

*Drafting of the manuscript*: Tzortzakakis, Papathomas, Sun.

*Critical revision of the manuscript for important intellectual content*: Tzortzakakis, Papathomas, Sun, Bazarova, Trpkov, Bozoky, Wang, Kokaraki, Arvanitis, Axelsson, Walch.

*Statistical analysis*: Bazarova.

*Obtaining funding*: None.

*Administrative, technical, or material support*: Kokaraki, Arvanitis.

*Supervision*: Tzortzakakis.

*Other*: None.

  ***Financial disclosures:*** Antonios Tzortzakakis certifies that all conflicts of interest, including specific financial interests and relationships and affiliations relevant to the subject matter or materials discussed in the manuscript (eg, employment/affiliation, grants or funding, consultancies, honoraria, stock ownership or options, expert testimony, royalties, or patents filed, received, or pending), are the following: Axel Walch reports grants from Deutsche Forschungsgemeinschaft (SFB 824 C04, CRC/Transregio 205/1).

  ***Funding/Support and role of the sponsor:*** This study was financially supported by VINNOVA (MIDOR study, reference number 2015-0180).

## CRediT authorship contribution statement

**Thomas Papathomas:** Conceptualization, Data curation, Formal analysis, Investigation, Methodology, Project administration, Resources, Software, Validation, Visualization, Writing - original draft, Writing - review & editing. **Antonios Tzortzakakis:** Conceptualization, Data curation, Formal analysis, Investigation, Methodology, Project administration, Resources, Software, Supervision, Validation, Visualization, Writing - original draft, Writing - review & editing. **Na Sun:** Data curation, Formal analysis, Investigation, Methodology, Project administration, Resources, Software, Supervision, Validation, Visualization, Writing - original draft, Writing - review & editing. **Franziska Erlmeier:** Data curation. **Annette Feuchtinger:** Data curation. **Kiril Trpkov:** Data curation, Formal analysis, Investigation, Methodology, Project administration, Resources, Software, Writing - review & editing. **Alina Bazarova:** Formal analysis, Investigation, Methodology, Project administration, Resources, Software, Writing - review & editing. **Alexandros Arvanitis:** Formal analysis, Investigation, Methodology, Project administration, Resources, Software, Writing - review & editing. **Wanzhong Wang:** Formal analysis, Investigation, Methodology, Project administration, Resources, Software, Writing - review & editing. **Bela Bozoky:** Formal analysis, Investigation, Methodology, Project administration, Resources, Software, Writing - review & editing. **Georgia Kokaraki:** Investigation, Methodology, Project administration, Resources, Software, Writing - review & editing. **Rimma Axelsson:** Conceptualization, Formal analysis, Funding acquisition, Investigation, Methodology, Project administration, Resources, Software, Writing - review & editing. **Axel Walch:** Conceptualization, Formal analysis, Funding acquisition, Investigation, Methodology, Project administration, Resources, Software, Writing - review & editing.
